# Addition of Selected Plant-Derived Proteins as Modifiers of Inulin Hydrogels Properties

**DOI:** 10.3390/foods9070845

**Published:** 2020-06-29

**Authors:** Anna Florowska, Adonis Hilal, Tomasz Florowski, Małgorzata Wroniak

**Affiliations:** Institute of Food Science, Department of Food Technology and Assessment, Warsaw University of Life Sciences-SGGW, 159c Nowoursynowska Street, 02-787 Warsaw, Poland; adonis.hilal@gmail.com (A.H.); tomasz_florowski@sggw.edu.pl (T.F.); malgorzata_wroniak@sggw.edu.pl (M.W.)

**Keywords:** inulin hydrogel, pea protein, soy protein

## Abstract

The aim of the study was to determine the effects of pea and soy protein addition (1, 3, 6 g/100 g) on inulin hydrogels properties. Inulin hydrogels (20 g/100 g) were obtained by thermal induction. It was stated that tested plant protein might be used as a modifier of inulin hydrogels properties. The addition of pea and soy protein to inulin hydrogels resulted in networks with more a compact and homogeneous structure. The increase of the protein concentration caused the structure of the hydrogels to get smoother, more cohesive, and less granular. Pea and soy protein addition (3–6 g/100 g) to hydrogels allowed to obtain higher values of yield stress, texture (firmness, adhesiveness) and spreadability parameters. At a protein concentration of 6 g/100 g, the firmness of inulin hydrogels was seven times higher for those with pea protein (1.87 N) and ten times higher for those with soy protein (2.60 N) compering to the control hydrogel (0.24 N). The transmission profiles of hydrogels with incorporated 6 g/100 g of soy proteins showed the slowest motion of the particles, which indicates the highest stability of gel. As the concentration of protein addition increased, a reduction in the lightness was observed.

## 1. Introduction

Inulin is well known prebiotic polysaccharide (molecular weight around 5 kDa) constituted of fructose molecules linked by β-(2/1)-D-fructosyl-fructose bonds of various length, terminated generally by a single glucose molecule linked by an α-D- glucopyranosoyl bond [1,2]. By conjugating this type of prebiotic polysaccharide to the protein it is possible to create a new generation of prebiotic ingredients. Proteins and their amino acids are known to be an important nitrogen source for bacteria, however, they are scarce in the colon due to their absorption in the small intestine. That is why protein-prebiotic carbohydrate complexes are expected to form self-assembling micellar particles, wherein the indigestible carbohydrate units will protect core protein units from digestive enzymes, in consequences making proteins indigestible and nonabsorbable and possibly available to be used by probiotic bacteria in the colon [3]. Polysaccharides as cover materials for encapsulation of proteins and peptides are already being used for targeted anti-cancer drug delivery [2,4,5]. The properties of the protein–polysaccharide complex depend on characteristics of both biopolymers (such as molecular parameters of each biopolymer: weight and conformation, charge density and rigidity), as well as their concentrations, and medium conditions (such as pH, ionic strength, temperature, etc.). The protein–polysaccharide complex can behave in different ways: one is co-solubility, which occurs at low biopolymer concentration or in mixtures of small or intermediate molecular-sized biopolymers; the second is thermodynamic incompatibility (segregative interactions), or complex coacervation (aggregative interactions). Both thermodynamic incompatibility and complex coacervation involve physical and chemical forces with a variety of charge, magnitude, strength, and direction [6,7]. That complex formation happens either by enthalpy or entropy, depending on the charge density of macromolecules. Weakly charged proteins and polysaccharides complexes arise as a result of electrostatic interactions (enthalpic contribution) but the formation of aggregated complexes or aggregates is often entropically driven, probably by counterions and water molecules release and conformational changes of proteins and/or polysaccharides [8].

Investigating the properties of such protein-prebiotic carbohydrate complexes is very important since they both have gelling properties and contribute to food stability, texture, sensation, and shelf life [9]. It is also well known that proteins and polysaccharides affect the gelling properties of each other and these interactions might have a positive [10,11,12] as well as a negative influence [13,14] on gel structure. Interactions between proteins and polysaccharides may occur physically through electrostatic interactions or chemically through covalent bonds [15]. Noncovalent interactions can be grouped as follows, either, attractive (i.e., hydrophobic, hydrogen bonds, Van der Waals, disulfide bonds) or repulsive (i.e., electrostatic, hydration, steric repulsions) [16]. Covalent interactions are highly specific, causing permanent binding and irreversible interaction between proteins and polysaccharides [17].

Colloidal particles formed out of proteins and polysaccharides might have applications in a variety of food products as stabilizers in emulsions [18,19] as foaming improvements [20,21] or for their rheological properties [10]. That is why those colloidal particles are now very often used in developing novel, functional food products, e.g., high in protein or with reduced fat content. According to O’Chiu and Vardhanabhuti [21], polysaccharide-whey protein isolate complex might be used to modify the properties of aerated dairy gels, to create novel-textured high-protein desserts, where aeration can increase their attractiveness by innovative textures and reduced calories by volume. Applying polysaccharide-milk protein colloidal into low-fat ice-creams might solve, a very common for low-fat ice-creams, textural problems such as coarseness and iciness, crumbly body, shrinkage as well as flavor defects [22,23]. Furthermore, in low-fat meat products, incorporating a polysaccharide-whey protein isolate (WPI) complex might bring benefits such as higher stability and water retention as well as acceptable sensory scores [24].

Often in food products, the protein component of the protein–polysaccharide complex is animal-origin. Very popular are milk proteins, such as whey isolates or sodium caseinate. Currently in the production of new and high-quality food products natural plant-derived proteins are being used. This change might be due to the growth of consumers’ interest in the vegan lifestyle as well as anxiety related to food allergies [25,26] and higher prices of animal-derived protein products [27]. The plant-based proteins mainly used in the food industry are derived from wheat and soybean [28]. Soy protein isolate is obtained from defatted flakes or soy flour by extraction with dilute alkali (pH 8–9) followed by centrifugation to remove insoluble materials. Next the acidification (pH 4.5) with a food grade acid is used to selective recovery of the proteins [29]. To improve the functional properties of soy protein isolates membrane technologies, such as ultrafiltration and electro-acidification, are being used [30]. Soy protein isolate contains at least 90 g/100 g protein, dominated by two major storage (globulin) proteins: beta-conglycinin (7S globulin, 150 kDa) and glycinin (11S globulin, 350–400 kDa) [31]. Beta-conglycinin is a trimeric protein composed of three subunits (α’, α and β). Glycinin is a hexameric protein composed of five subunits A_1a_B_1b_,A_2_B_1a_,A_1b_B_1a_,A_5_A_4_B_3_, and A_3_B_4_, and each subunit is composed of acidic and basic polypeptides, which are linked together by a disulfide bond [32]. Soy protein isolate (SPI) is a commonly used vegetable protein for its good water and fat holding capacity, excellent gelling and structuring properties [33] as well as for non-toxic nature, low cost, with high nutritional values, and its Generally Regarded As Safe (GRAS) status [34]. In the past decade, there has also been an interest in the commercial development of products with proteins from legumes, in particular pea protein [35,36,37,38,39]. Pea protein is usually a by-product of the pea starch industry [40] and can be derived from plant material [41] by wet processing, in which proteins are isolated by a selective precipitation step at the isoelectric point [42]. Pea protein isolates has usually a protein content of 85 g/100g with the presence of salt-soluble globulins and water-soluble albumin proteins. The content of globulins is 70–80% whereas that of albumin is 10–20% of the total proteins [43]. Among globulins, legumin (11S), vicilin (7S), and convicilin (7S) are the predominant ones. The 11S legumin is a protein with a hexameric quaternary structure (molecular weight around 320–380 kDa) consists of an acidic and a basic polypeptide linked together by a disulfide bridge. The 7S vicilin (150–180 kDa) and 7S convicilin (210–290 kDa) are proteins with trimer quaternary structure [44,45]. Pea protein is valuable for its balanced amino acid composition, but also prominent lysine content [40], and what is more, pea protein isolate is not listed as an allergenic ingredient and less connected to GMO questions [41,46]. Pea protein also has good dispersion, stability, fluidity and other water-soluble characteristics, good foaming and emulsifying properties, as well as good gelling properties [47,48,49,50]. In the available literature, there are not many pieces of researches that focus on the properties of plant protein–polysaccharide complexes. Therefore, the objective of this study was to investigate the effects of pea and soy protein addition on gelation properties of inulin in creating a new complex of hydrogels that might be used in novel, functional food products.

## 2. Materials and Methods 

### 2.1. Materials

Inulin Orafti^®^ HPX (average degree of polymerization DP ≥ 23) purchased from BENEO GmbH (Mannheim, Germany). Plant protein: pea protein isolate, soy protein isolate, purchased from MYPROTEIN (Cheshire, United Kingdom).

### 2.2. Induction of Inulin Hydrogels

Hydrogels were formulated using the thermal method. The first step was to dissolve inulin (20 g/100 g) in water (80 °C) using a heating magnetic stirrer (1500 RPM for 5 min). Then the resulting solution was cooled to 45 °C and selected proteins (pea protein isolate or soy protein isolate) at concentrations 1, 3, and 6 g/100 g were added and mixed again using a magnetic stirrer (1500 RPM for 5 min). Solutions were kept (at 8 °C) for 24 h to form hydrogel’s structure. After this time, the formation of inulin hydrogels was analyzed, as well as the microstructure, textural properties, yield stress, stability, and color parameters. 

### 2.3. Methods

#### 2.3.1. Volumetric Gel Index (VGI)

The VGI was used to analyze the degree of inulin hydrogel formation. It is a parameter to express the ability to form a gel structure. In the case where the gel structure is not formed VGI is equal to 0, and for the completely gelled sample VGI will equal to 100%. To calculate VGI the glass container (ø 50 mm, height 50 mm) was filled with inulin or inulin–protein solution and after 24 h of storage (at 8 °C) the gel network was formed. After that time, the total volume of the samples (VT) and the volume of formed gels were measured (VG). 

The volumetric gel index (VGI) was expressed as [51]:

$$VGI=\frac{VG}{VT}\times 100\;\left[\%\right]$$

where VG—volume of formulated gel and VT—total volume of sample.

#### 2.3.2. Microstructure

An electron scanning microscope (FEI Quanta 200 ESEM, Hillsboro, Oregon, USA) equipped with an energy dispersive spectrometer (EDS) and digital image recording was used to determine the microstructure of inulin and inulin-protein hydrogels. A small amount of a freeze-dried inulin hydrogel was applied to a carbon band and coated with a thin layer of gold, and observed at pressures of 100–133 Pa, under an accelerating voltage of 25 or 30 kV. The graphical elaboration of the gel structure was performed using MultiScan v.18.03 software (Computer Scanning System, Warsaw, Poland).

#### 2.3.3. Textural Properties

Texture analysis was determined using a texture analyzer (TA.XT Plus, Stable Micro Mixtures, Surrey, UK) with a 5 kg load cell at 20 °C. For measurement of the firmness (N) and adhesiveness (Ns), the texture analyzer was equipped with a 0.5-cm diameter cylindrical flat probe (P/0.5R). The speed of measurements was 1.0 mm/s and the trigger force was 1 g. Samples were prepared in cylindrical containers (ø 50 mm, height 50 mm) and the penetration depth of the inulin hydrogel was 8 mm. For spreadability measurement, the texture analyzer was equipped with TTC Spreadability Rig. The speed of measurement and distance was 3.0 mm/s and 20 mm, respectively. The reported values represent the averages of six replicates. The data were analyzed using the Exponent version 6.1.4.0 equipment software. The software of the instrument recorded a force/displacement curve, measuring positive as well as negative values. Three parameters were measured: maximum force, negative maximum force and negative area, corresponding to firmness, spreadability and adhesiveness, respectively.

#### 2.3.4. Yield Stress

To measure the yield stress of obtained hydrogels a rheometer (DV3T, Brookfield, Middleboro, MA, USA) was used. Measurements were conducted at 20 °C using spindles dedicated to yield stress (Pa) analysis: vane spindle V74 with a torque range HA. Samples were prepared in cylindrical containers (ø 50 mm, height 50 mm) and the test speed was 0.10 RMP. The reported values represent the averages of six replicates. The values of texture attributes were analyzed using the software provided with the rheometer.

#### 2.3.5. Physical Stability

The physical stability of inulin hydrogels was determined with an analytical centrifuge—LUMiSizer 6120-75 (L.U.M. GmbH, Berlin, Germany) by measuring the intensity of transmitted near-infrared light in suspension. Stability was shown as space and time-related transmission profile over the sample length. The parameters used for the analysis were: wavelength 870 nm, volume 1.8 mL of dispersion; light factor: 1; 1500 rpm; experiment time, 15 h 10 min; interval time 210 s; temperature 20 °C. The reported values represent the average of six replicates. The data were analyzed by the delivered software (SepView 6.0; LUM, Berlin, Germany) and the instability index was calculated.

#### 2.3.6. Color Parameters 

To determine the color components (L*, a*, and b*) the Minolta CR-200 colorimeter (Minolta, Japan; light source D65, observer 2°, a measuring head hole of 8 mm) was used. Color parameters were analyses using the CIEL*a*b* system. The measurements were made at the surface of inulin’s hydrogel. To determine the color differences between control hydrogels and gels with protein addition, the parameter of total color difference ΔE was calculated [52].

$$\Delta E=\sqrt{{\left(L_{c}^{*}-L_{P}^{*}\right)}^{2}+{\left(a_{c}^{*}-a_{P}^{*}\right)}^{2}+{\left(b_{c}^{*}-b_{P}^{*}\right)}^{2}}$$

where ${\;L}_{c}^{*},{\;a}_{c}^{*},b_{c}^{*}$ refers to the colour parameters of control hydrogels without protein addition and $L_{P}^{*},{\;a}_{P}^{*},b_{P}^{*}$ refers to the colour parameters of hydrogels with pea or soy protein addition.

#### 2.3.7. Statistical Analysis

The results, collected from three experimental replicates produced at separate times, were statistically analyzed using Statistica 13.3 (TIBCO Software Inc. Palo Alto, CA, USA) program. To determine the significance of differences between the average values of analyzed parameters of inulin hydrogels with soy and pea protein one-way analysis of variance was used. Significant differences between inulin hydrogels without and with different proteins (soy or pea) concentrations were verified using Tukey’s test at a significant level α = 0.05.

## 3. Results and Discussion

### 3.1. Effect of Soy and Pea Protein Addition on Gel Formation Ability and Microstructure of Inulin Hydrogels

It was found that the addition of both pea and soy proteins, independently from its concentration, has not affected the gel formation ability. Under investigated conditions (20 g/100 g of inulin, 1, 3, 6 g/100 g of protein respectively) the inulin-plant protein water suspension formed a homogeneous gel structure (VGI = 100 %, Table 1).

**Figure 1:**
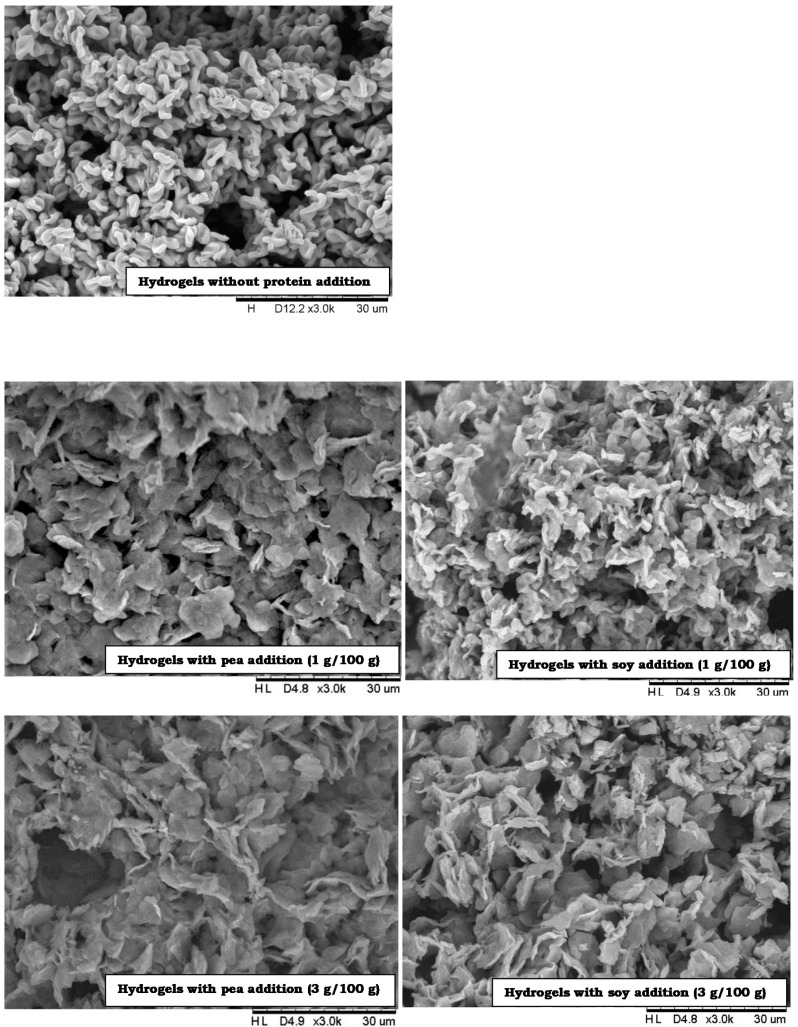

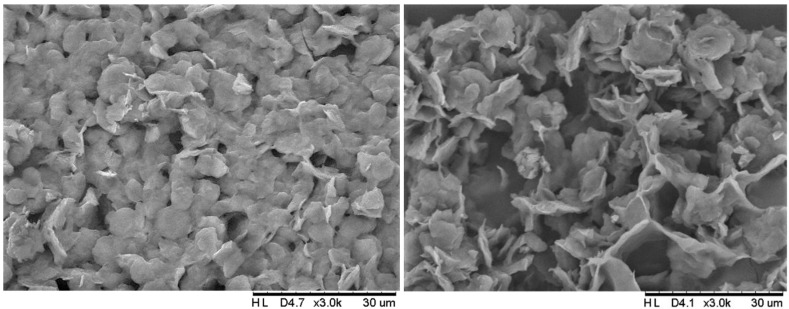
Influence of pea and soy protein addition on inulin hydrogel structure.

To observe the influence of the addition of soy and pea protein on the microstructure of inulin hydrogels electron microscopy was used (Figure 1). Inulin hydrogels had a sponge-like appearance. The incorporation of soy and pea protein to the inulin hydrogels caused a visible modification of the structure. Hydrogels with protein addition were compact and more homogeneous. Moreover, the protein increment increased the smoothness, cohesiveness and decreased the granular gel appearance and structure of the gel. The same observation was made by Glibowski [12] who investigated the influence of whey protein on the structure of inulin gels. The author has noticed that the structure of whey-inulin gels was thicker. Furthermore, Picone, Maximo, Kuhn, Ros-Polski, and Cunha [53] claimed that samples with inulin and caseinate gels appeared to have slightly more closed pores than the pure protein samples. Compering the studies of Turgeon et al. [54] with the studies conducted for the purposes of this publication, and by analyzing the SEM images, it was found that hydrogels with the addition of plant proteins did not show the phenomenon of thermodynamic incompatibility, in other words, no repulsion or lack of interaction between protein and inulin molecules were observed. However, a visible compact structure may indicate the phenomenon of thermodynamic compatibility caused by the existence of electrostatic attraction between these two biopolymers.

### 3.2. Effect of Plant Protein Addition on Textural Properties and Yield Stress of Inulin Hydrogels

Analyzing the texture measurement results, a significant effect of the addition (3, 6 g/100 g) of both pea and soy proteins on the firmness of inulin hydrogels was found. With the higher concentration of protein, the force needed to cause deformation of the hydrogels increased, which means that the hydrogels with the addition of selected proteins were harder (Table 1). At soy protein addition on level of 6 g/100 g, the firmness of the tested hydrogels was higher than that of adequate levels of pea proteins. At this protein concentration, the firmness of inulin hydrogels was seven times higher for pea proteins (1.87 N) and ten times higher for soy proteins (2.60 N) compering to inulin hydrogels without the protein addition (0.24 N). Soy protein consists of globulins containing glycinin fraction (glycinin and β-conglycinin), which improves gel formation capacity. In particular, glycinin introduces hardness and turbidity of gels whereas β-conglycinin is responsible for the elasticity of thermally induced gels [55]. The influence of protein addition on the firmness of hydrogels might be due to a more compact structure that was visible in microstructural patterns in SEM (Figure 1). Synergistic interactions between protein and inulin on textural properties were also observed by Glibowski [12] during the effect examination of the ratio of whey proteins and inulin on the properties of the obtained hydrogels. The addition of proteins to inulin hydrogels increases their firmness by increasing the attraction forces between protein molecules, which is confirmed by the studies on the interaction of inulin and whey proteins conducted by Buriti et al. [56], Glibowski et al. [57] and Akal, Karagözlü, and Ünal [58].

In the case of adhesiveness, it was found that at lower levels (1 and 3 g/100 g) of the soy and pea proteins concentration the adhesion of the inulin-protein hydrogels did not differ compared to hydrogels without protein addition (Table 1). This could be a consequence of the formation of stronger bonds between inulin crystals than between inulin crystals and proteins. When protein concentration in inulin-protein hydrogels was higher (6 g/100 g) the induced hydrogels had significantly higher adhesiveness than control hydrogels. Additionally, it was found that soy protein had influenced the adhesiveness more than pea protein. Adhesion between gel microparticles is probably due to the formation of thin liquid bridges between their surfaces [59]. As hydrogels are water-swollen polymeric materials that maintain a distinct three-dimensional structure [60] they are very often used as biopolymers fat-mimetics [61] for which adhesiveness is along with spreadability one of the major quality discriminants. The spreadability of inulin hydrogels (Table 1), expressed as the force needed to destroy the gel structure during spreading was the highest for hydrogels containing 6 g/100 g of plant-protein concentration (21.74 N for soy, and 9.23 N for pea), the same correlation was observed in case of firmness. Additionally, it was found that soy protein had a greater impact on the spreadability than pea protein. Van Der Berg et al. [62] in their research covering the physical properties of protein–polysaccharide hydrogels stated that the firmness of the hydrogels depends on their deformability, and the spreadability relates to the number of deformed and separated structures.

For the characterization of the influence of pea and soy protein on the resistance of inulin hydrogels to flow under stress, the yield stress was analyzed (Table 1). It was reported that yield stress is influenced by protein concentration. An addition of pea protein (at concentration 1–6 g/100 g), as well as soy protein (3–6 g/100g), caused the formation of hydrogels with significantly higher resistance to flow under stress compared to the control sample. The globular proteins suspended in water constitute a colloidal suspension [63]. Pea and soy protein gives Newtonian dispersions in medium-range concentrations (up to 6.0 g/100 g) [64]. Aqueous colloids of globular protein have been shown to exhibit solid-like mechanical responses to oscillating small strains and yield stress against flow [65]. It was stated that the yield stress of inulin-protein hydrogels depends on protein concentration, higher protein concertation resulted in higher yield stress. It was found that in the case of soy protein addition (in the amount of 3 and 6 g/100 g) the structure of the hydrogels was stronger (higher yield stress values: 564.7 and 1746.0 Pa respectively) in comparison to hydrogels with the pea protein addition (448.0 and 1644.7 Pa respectively) at the same concentration. The increased value in the yield stress after plant protein addition may be triggered by the hydration of the added proteins, which contributes changes in the properties of the hydrogels [66]. It is well known that interactions between polysaccharide and protein molecules are favored, therefore polysaccharide adsorption may occur leading to steric stabilization of protein micelles [10].

### 3.3. Effect of Pea and Soy Protein Addition on Stability of Inulin Hydrogels

**Figure 2:**
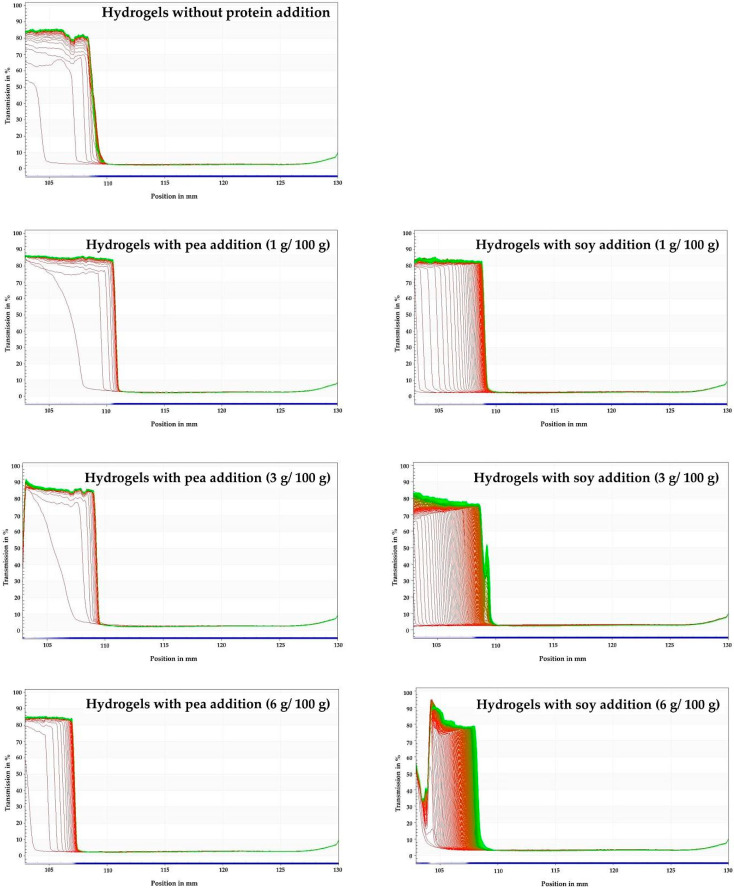
Influence of pea and soy protein addition on inulin hydrogel transmission profiles presented enabling LUMiSizer^®^ analysis.

Inulin hydrogels were traced as space- and time-related transmission profile over the samples by using the LUMiSizer^®^ (Figure 2). Observing the changes in light transmission through the samples, the instability that can occur in the tested hydrogels can be seen [67]. All tested hydrogels showed a similar shape of the transmission profile, which indicates the movement of the particles towards the bottom of the sample, as a result of particle sedimentation process. Based on the course of transmission profiles, it can be stated that the sedimentation process took place the fastest in the control sample, as well as in hydrogels with pea protein. In inulin hydrogels with the addition of soy proteins, the movement of particles was at a slower rate than in the case of hydrogels containing pea proteins and the control sample. The higher the soy protein concentration, the slower the changes occurred. 

**Figure 3:**
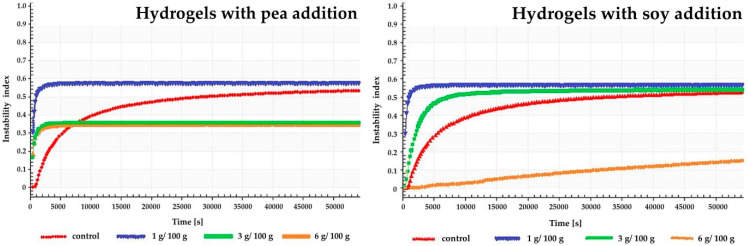
Influence of pea and soy addition on stability inulin hydrogels.

The gel profile with the addition of 6 g/100 g soy proteins showed the slowest motion of the particles, which indicates the highest stability of the gel. The stability of hydrogels was also presented as the instability index (Table 1, Figure 3). The higher the index value was, the less stable the colloidal dispersion was. It was noted that the addition of 1 and 3 g/100 g of soy protein and 1 g/100 g of pea protein did not affect the instability index values of inulin hydrogels. On the other hand, the addition of 3 and 6 g/100 g of pea proteins reduced the value of the index (from 0.53 for pure inulin hydrogels to 0.35 and 0.36 respectively for pea addition), what can be concluded that the addition of pea proteins at these levels increased the stability of inulin hydrogels. The gel with the 6 g/100 g of soy protein addition had the highest stability seeing that the instability index reached the lowest value (0.15). Although in the literature there is no information regarding the stabilization of inulin gels by incorporating the protein, there are studies on whey protein mixtures with inulin in which was found that the presence of inulin in solutions of whey protein caused an increase in viscosity as well as stability of the probes [68]. Furthermore, Tseng et al. [69] showed that inulin can stabilize native soy proteins gel and improve gel structure.

### 3.4. Effect of Pea and Soy Protein Addition on Colour Parameters of Inulin Hydrogels

The addition of plant proteins to inulin hydrogels significantly decreased the color component value L* (Table 2). As the concentration of protein addition increased, the value of this parameter decreased, leading to a decrease of lightness. With the increase in the concentration of incorporated protein, the value of the L* parameter decreased from 87.89 for the control sample to 79.04 for the sample with the 6 g/100 g of pea proteins addition and 80.27 for 6 g/100 g of soy protein.

A similar trend was observed for the value of the a* color parameter, which increased significantly with the increase in the level of plant protein addition (Table 2). Pea proteins had a greater impact on changes in the a* parameter of the hydrogels when comparing to soy proteins. An almost threefold increase in the studied parameter was observed at a 6 g/100 g concentration of pea proteins (1.34) over inulin hydrogels without added protein (−0.73). Based on the obtained results, an effect on the b* color parameter was found, due to the increased concentration of plant protein addition (Table 2). Which is a phenomenon confirmed by the literature data [70]. In the case of the addition of 1 and 3 g/100 g of pea proteins, the increase in the value of the tested parameter was lower than in the case of the same concentrations of soy protein. However, for the addition of 6 g/100 g of soy protein as well as pea protein, the obtained value of the b * parameter did not differ (12.40 and 12.55 respectively). The addition of pea or soy protein into food products also results in a decrease in L* values and an increase in a* and b* parameters [71,72,73]. In the interest of a comprehensive analysis of the effect of pea and soy protein addition on the color of inulin gels, the total color difference parameter (ΔE) was calculated. It has been determined that the color difference between the control inulin hydrogel and gels with the lowest protein concertation (1 g/100g) was noticed even by the inexperienced observer (2 < ΔE < 3.5). Higher (3–6 g/100 g) concentration of proteins has caused observers to recognize two different inulin gels colors compared to the control inulin gels (5 < ΔE) (Table 2) [52].

## 4. Conclusions

The addition of both pea and soy proteins did not affect the formation of inulin hydrogels. Despite that, the properties of the obtained hydrogels were strongly affected by that protein addition. They caused the microstructure of hydrogels to become more compact and homogeneous. With the increase of protein concentration, the gel structure was smoother, more cohesive, and less granular. As a result, hydrogels with the addition of plant proteins (3–6 g/100g) had significantly (α = 0.05) higher rheological parameters value, especially firmness, yield stress, and spreadability. Additionally, the stability of hydrogels increased with the increase of the concertation of added plant proteins. Hydrogels with 6 g/100 g of soy protein addition manifested the highest stability. The incorporation of the protein resulted in a visible color change. The increasing level of both pea and soy proteins led to a reduction in the lightness of inulin hydrogels as well as an increase in the b* parameter value. Based on the achieved results, it can be stated that by the addition of protein into inulin hydrogels gels, new technological properties can be obtained.

## Figures and Tables

**Table 1:** Physical properties of inulin hydrogels obtained without or with the addition of pea and soy proteins.

	Concertation of Protein [g/100 g]	VGI [%]	Firmness [N]	Adhesiveness [Ns]	Spreadability [N]	Yield Stress [Pa]	Instability Index
Control	0	100 ^a^	0.24 ^a^ ± 0.02	−0.10 ^a^ ± 0.01	2.43 ^a^ ± 0.12	195.0 ^a^ ± 8.0	0.53 ^c^ ± 0.04
Pea protein	1	100 ^a^	0.35 ^ab^ ± 0.12	−0.11 ^a^ ± 0.00	2.45 ^a^ ± 0.44	271.3 ^b^ ± 26.5	0.57 ^c^ ± 0.03
	3	100 ^a^	0.56 ^bc^ ± 0.14	−0.17 ^a^ ± 0.00	3.38 ^b^ ± 0.27	448.0 ^c^ ± 25.5	0.36 ^b^ ± 0.09
	6	100 ^a^	1.87 ^d^ ± 0.09	−0.44 ^b^ ± 0.04	9.23 ^c^ ± 0.20	1644.7 ^e^ ± 34.4	0.35 ^b^ ± 0.01
Soy protein	1	100 ^a^	0.47 ^abc^ ± 0.07	−0.13 ^a^ ± 0.01	3.55 ^b^ ± 0.09	237.3 ^ab^ ± 24.1	0.56 ^c^ ± 0.01
	3	100 ^a^	0.76 ^c^ ± 0.02	−0.17 ^a^ ± 0.02	6.00 ^c^ ± 0.04	564.7 ^d^ ± 7.0	0.54 ^c^ ± 0.02
	6	100 ^a^	2.60 ^e^ ± 0.20	−1.26 ^c^ ± 0.16	21.74 ^e^ ± 0.28	1746.0 ^f^ ± 38.0	0.15 ^a^ ± 0.03

Values are mean ± SD (*n* = 3). a–f—values followed by the same letter within a column do not differ significantly according to Turkey’s test (*p* < 0.05).

**Table 2:** Color parameters and the total color difference parameter of inulin hydrogels obtained without or with the addition of pea and soy proteins.

	Concertation of Protein [g/100 g]	Color Parameters			ΔE ^#^
		L*	a*	b*	
Control	0	87.89 ^a^ ± 1.45	−0.73 ^a^ ± 0.33	2.50 ^a^ ± 0.35	-
Pea protein	1	86.22 ^b^ ± 0.07	−0.24 ^b^ ± 0.03	3.65 ^b^ ± 0.04	2.18 ± 0.77
	3	82.38 ^c^ ± 0.15	0.68 ^c^ ± 0.01	9.40 ^d^ ± 0.21	8.97 ± 0.96
	6	79.04 ^d^ ± 0.05	1.34 ^d^ ± 0.01	12.55 ^f^ ± 0.03	13.56 ± 0.86
Soy protein	1	86.29 ^b^ ± 0.05	−0.83 ^a^ ± 0.11	5.43 ^c^ ± 0.01	3.40 ± 0.67
	3	80.78 ^c^ ± 0.09	−0.26 ^b^ ± 0.13	9.85 ^e^ ± 0.06	10.25 ± 0.90
	6	80.27 ^c^ ± 0.17	−0.03 ^b^ ± 0.01	12.40 ^f^ ± 0.13	12.54 ± 0.82

Values are mean ± SD (*n* = 3). a–f—values followed by the same letter within a column do not differ significantly according to Tukey’s test (*p* < 0.05). ^#^ total color difference parameter calculated in relation to the control sample.

## References

[1] Zhao M., Mu W., Jiang B., Zhou L., Zhang T., Lu Z., Zhengyu J., Yang R. (2011). Bioresource Technology Purification and characterization of inulin fructotransferase ( DFA III-forming ) from *Arthrobacter aurescens* SK 8. 001. Bioresour. Technol..

[2] Florowska A., Krygier K., Florowski T., Dłużewska E. (2016). Prebiotics as functional food ingredients preventing diet-related diseases. Food Funct..

[3] Seifert A., Freilich S., Kashi Y., Liveney Y.D. (2019). Protein oligosaccharide conjugates as novel prebiotics. Polym. Adv. Technol..

[4] Dheer D., Arora D., Jaglan S., Rawal R.K., Shankar R. (2017). Polysaccharides based nanomaterials for targeted anti-cancer drug delivery. J. Drug Target..

[5] Zhang L., Pan J., Dong S., Li Z. (2017). The application of polysaccharide-based nanogels in peptides/proteins and anticancer drugs delivery. J. Drug Target..

[6] Ghosh A.K., Bandyopadhyay P., Karunaratne D.N. (2012). Polysaccharide-Protein Interactions and Their Relevance in Food Colloids. The Complex World of Polysaccharides.

[7] Behrouzain F., Razavi S.M.A., Joyner H. (2020). Mechanisms of whey protein isolate interaction with basil seed gum: Influence of pH and protein–polysaccharide, ratio. Carbohydr. Polym..

[8] Turgeon S.L., Schmitt C., Sanchez C. (2007). Protein – polysaccharide complexes and coacervates. Curr. Opin. Colloid Interface Sci..

[9] Herceg Z., Rezek A., Lelas V., Kresić G., Franetović M. (2007). Effect of carbohydrates on the emulsifying, foaming and freezing properties of whey protein suspensions. J. Food Eng..

[10] Paraskevopoulou A., Athanasiadis I., Blekas G., Koutinas A.A., Kanellaki M., Kiosseoglou V. (2003). Influence of polysaccharide addition on stability of a cheese whey kefir-milk mixture. Food Hydrocoll..

[11] Tavares C., Silva J.A.L. (2003). Rheology of galactomannan—Whey protein mixed systems. Int. Dairy J..

[12] Glibowski P. (2009). Rheological properties and structure of inulin-whey protein gels. Int. Dairy J..

[13] Shim J., Mulvaney S.J. (2001). Effect of heating temperature, pH, concentration and starch / whey protein ratio on the viscoelastic properties of corn starch / whey protein mixed gels. J. Sci. Food Agric..

[14] Patino R.J.M., Pilosof M.R.A. (2011). Protein–polysaccharide interactions at fluid interfaces. Food Hydrocoll..

[15] Dickinson E. (2008). Interfacial structure and stability of food emulsions as affected by protein–polysaccharide interactions. Soft Matter.

[16] Bryant C.M., Mc Clements D.J. (1998). Molecular basis of protein functionality with special consideration of cold-set gels derived from heat- denatured whey. Trends Food Sci. Technol..

[17] Schmitt C., Sanchez C., Desobry-Banon S., Hardy J. (1998). Structure and Technofunctional Properties of Protein–polysaccharide, Complexes: A Review. Crit. Rev. Food Sci. Nutr..

[18] Zang X., Wang J., Yu G., Cheng J. (2019). Addition of anionic polysaccharides to improve the stability of rice bran protein hydrolysate-stabilized emulsions. LWT Food Sci. Technol..

[19] Zhang H., Fan Q., Li D., Chen X., Liang L. (2019). Impact of gum Arabic on the partition and stability of resveratrol in sunflower oil emulsions stabilized by whey protein isolate. Colloids Surf. B Biointerfaces.

[20] Neves I.C.O., de Faria J.T., Vidigal M.C.T.R., Fidelis P.C., Minim V.P.R., Minim L.A. (2018). Foaming properties of suspensions composed by β-lactoglobulin and polysaccharides, in the presence of sucrose or polyols. Colloids Surf. A Physicochem. Eng. Asp..

[21] O’Chiu E., Vardhanabhuti B. (2017). Utilizing whey protein isolate and polysaccharide complexes to stabilize aerated dairy gels. J. Dairy Sci..

[22] Cheng J., Ma Y., Li X., Yan T., Cui J. (2015). Effects of milk protein–polysaccharide, interactions on the stability of ice cream mix model systems. Food Hydrocoll..

[23] Schaller-Povolny L., Smith D.E. (2002). Interaction of milk proteins with inulin. Milchwissenschaft.

[24] Andrès S., Zaritzky N., Califano A. (2006). The effect of whey protein concentrates and hydrocolloids on the texture and color characteristics of chicken sausages. Int. J. Food Sci. Technol..

[25] Adebiyi A.P., Aluko R.E. (2011). Functional properties of protein fractions obtained from commercial yellow field pea (*Pisum sativum L.*) seed protein isolate. Food Chem..

[26] Toews R., Wang N. (2013). Physicochemical and functional properties of protein concentrates from pulses. Food Res. Int..

[27] Chavan U.D., Mckenzie D.B., Shahidi F. (2001). Protein classification of beach pea (*Lathyrus maritimus* L.). Food Chem..

[28] Shevkani K., Singh N., Kaur A., Rana J.C. (2015). Structural and functional characterization of kidney bean and field pea protein isolates: A comparative study. Food Hydrocoll..

[29] Mounts T.L., Wolf W.J., Martinez W.H., Wilcox J.R. (1987). Processing and utilization. Soybeans: Improvement, Production, and Uses.

[30] Alibhai Z., Mondor M., Moresoli C., Ippersiel D., Lamarche F. (2006). Production of soy protein concentrates isolates: Traditional and membrane technologies. Desalination.

[31] Zhang H., Mittal G. (2010). Biodegradable protein-based films from plant resources: A review. Environ. Prog. Sustain. Energy.

[32] Renkema J.M.S., Knabben J.H.M., Van Vliet T. (2001). Gel formation by β -conglycinin and glycinin and their mixtures. Food Hydrocoll..

[33] Berghout J.A.M., Boom R.M., van der Goot A.J. (2015). Understanding the differences in gelling properties between lupin protein isolate and soy protein isolate. Food Hydrocoll..

[34] Wan Z.L., Wang L.Y., Yang X.Q., Wang J.M., Wang L.J. (2016). Controlled formation and stabilization of nanosized colloidal suspensions by combination of soy protein and biosurfactant stevioside as stabilizers. Food Hydrocoll..

[35] Du M., Xie J., Gong B., Xu X., Tang W., Li X., Li C., Xie M. (2018). Extraction, physicochemical characteristics and functional properties of Mung bean protein. Food Hydrocoll..

[36] Feyzi S., Milani E., Golimovahhed Q.A. (2018). Grass Pea (*Lathyrus sativus L.*) Protein Isolate: The Effect of Extraction Optimization and Drying Methods on the Structure and Functional Properties. Food Hydrocoll..

[37] Bajaj P.R., Tang J., Sablani S.S. (2015). Pea Protein Isolates: Novel Wall Materials for Microencapsulating Flaxseed Oil. Food Bioprocess Technol..

[38] Chao D., Jung S., Aluko R.E. (2018). Physicochemical and functional properties of high pressure-treated isolated pea protein. Innov. Food Sci. Emerg. Technol..

[39] Peng W., Kong X., Chen Y., Zhang C., Yang Y., Hua Y. (2016). Effects of heat treatment on the emulsifying properties of pea proteins. Food Hydrocoll..

[40] Klemmer K.J., Waldner L., Stone A., Low N.H., Nickerson M.T. (2012). Complex coacervation of pea protein isolate and alginate polysaccharides. Food Chem..

[41] Kowalczyk D., Baraniak B. (2011). Effects of plasticizers, pH and heating of film- forming solution on the properties of pea protein isolate films. J. Food Eng..

[42] Salunkhe S.S., Kadam D.K. (1989). Handbook of World Food Legumes: Nutritional Chemistry, Processing Technology, and Utilization.

[43] Liang H.N., Tang C.H. (2013). pH-dependent emulsifying properties of pea (*Pisum sativum L.*) proteins. Food Hydrocoll..

[44] Gatehouse J.A., Lycett W., Croy R.R.D., Boulter D. (1982). The post-translational proteolysis of the subunits of vicilin from pea (*Pisum sativum L*). Biochem. J..

[45] Reinkensmeier A., Bußler S., Schlüter O., Rohn S., Rawel H.M. (2015). Characterization of individual proteins in pea protein isolates and air classified samples. Food Res. Int..

[46] Lam A.C.Y., Can Karaca A., Tyler R.T., Nickerson M.T. (2018). Pea protein isolates: Structure, extraction, and functionality. Food Rev. Int..

[47] Aluko R.E., Mofolasayo O.A., Watts B.M. (2009). Emulsifying and foaming properties of commercial yellow pea (*Pisum sativum* L.) seed flours. J. Agric. Food Chem..

[48] Taherian A.R., Mondor M., Labranche J., Drolet H., Ippersiel D., Lamarche F. (2011). Comparative study of functional properties of commercial and membrane processed yellow pea protein isolates. Food Res. Int..

[49] Arntfield S.D., Maskus H.D. (2011). 9-Peas and other legume proteins. Handb. Food Proteins.

[50] Kowalczyk W., Gustaw M., Swieca B., Baraniak A. (2014). study on the mechanical properties of pea protein isolate films. J. Food Process. Preserv..

[51] Kim Y., Faqih M.N., Wang S.S. (2001). Factors affecting gel formation of inulin. Carbohydr. Polym..

[52] Mokrzycki W.S., Tatol M. (2011). Color difference ΔE—A survey. Mach. Graph. Vis..

[53] Picone C.S.F., Maximo G.J., Kuhn K.R., Ros-Polski V., Cunha R.L. (2011). An assessment of the texture of acidified sodium caseinate gels with added inulin using response surface methodology. Int. J. Dairy Technol..

[54] Turgeon S.L., Beaulieu M. (2001). Improvement and modification of whey protein gel texture using polysaccharides. Food Hydrocoll..

[55] Fukushima D. (2001). Recent progress in research and technology on soybeans. Food Sci. Technol. Res..

[56] Buriti F.C.A., Castro I.A., Saad S.M.I. (2010). Effects of refrigeration, freezing and replacement of milk fat by inulin and whey protein concentrate on texture profile and sensory acceptance of synbiotic guava mousses. Food Chem..

[57] Glibowski P. (2006). Effect of inulin on rheological properties of whey protein solutions. Acta Agrophysica.

[58] Akal Â., Karagözlü C., Ünal G. (2008). Rheological properties of reduced-fat and low-fat ice cream containing whey protein isolate and inulin. Eur. Food Res. Technol..

[59] Adhikari B., Howes T., Bhandari B.R., Truong V. (2003). In situ characterization of stickiness of sugar-rich foods using a linear actuator driven stickiness testing device. J. Food Eng..

[60] Kopeček J., Yang J. (2007). Hydrogels as smart biomaterials. Polym. Int..

[61] Tárrega A., Costell E. (2006). Effect of inulin addition on rheological and sensory properties of fat-free starch-based dairy desserts. Int. Dairy J..

[62] Van Den Berg L., Van Vliet T. (2008). Physical Properties Giving the Sensory Perception of Whey Proteins/Polysaccharide Gels. Food Biophys..

[63] Hunter R. (2001). Foundations of Colloid Science.

[64] Berli C.L.A., Deiber J.A., Añón M.C. (1999). Connection between rheological parameters and colloidal interactions of a soy protein suspension. Food Hydrocoll..

[65] Ikeda S., Nishinari K. (2001). Weak Gel-Type Rheological Properties of Aqueous Dispersions of Non aggregated k-Carrageenan Helices. J. Agric. Food Chem..

[66] Zha F., Dong S., Rao J., Chen B. (2019). Pea protein isolate-gum Arabic Maillard conjugates improves physical and oxidative stability of oil-in-water emulsions. Food Chem..

[67] Florowska A., Florowski T., Sokołowska B., Janowicz M., Adamczak L., Pietrzak D. (2020). Effect of high hydrostatic pressure on formation and rheological properties of inulin gels. LWT Food Sci. Technol..

[68] Tobin J.T., Fitzsimons S.M., Kelly A.L., Philip M., Auty M.A.E., Fenelon M.A. (2010). Microparticulation of mixtures of whey protein and inulin. International J. Dairy Technol..

[69] Tseng Y., Xiong Y.L., Boatright W.L. (2008). Effects of Inulin / Oligofructose on the Thermal Stability and Acid-Induced Gelation of Soy Proteins. J. Food Sci..

[70] Galus S., Mathieu H., Lenart A., Debeaufort F. (2012). Effect of modified starch or maltodextrin incorporation on the barrier and mechanical properties, moisture sensitivity and appearance of soy protein isolate-based edible films. Innov. Food Sci. Emerg. Technol..

[71] Sun W., Xiong Y.L. (2015). Stabilization of cooked cured beef color by radical-scavenging pea protein and its hydrolysate. LWT Food Sci. Technol..

[72] Parmar N., Singh N., Kaur A., Virdi A.S., Thakur S. (2016). Effect of canning on color, protein and phenolic profile of grains from kidney bean, field pea and chickpea. Food Res. Int..

[73] Pérez S., Matta E., Osella C., De la Torre M., Sánchez H.D. (2013). Effect of soy flour and whey protein concentrate on cookie color. LWT Food Sci. Technol..

